# Patient activation in Europe: an international comparison of psychometric properties and patients’ scores on the short form Patient Activation Measure (PAM-13)

**DOI:** 10.1186/s12913-016-1828-1

**Published:** 2016-10-12

**Authors:** Jany Rademakers, Helle Terkildsen Maindal, Aslak Steinsbekk, Jochen Gensichen, Katja Brenk-Franz, Michelle Hendriks

**Affiliations:** 1 NIVEL – Netherlands Institute for Health Services Research, PO Box 1568, 3500 BN Utrecht, Netherlands; 2 Maastricht University, CAPHRI School for Public Health and Primary Care, Maastricht, Netherlands; 3 Department of Public Health / Section of Health Promotion and Health Services Research, Aarhus University, Aarhus, Denmark; 4 Department of Public Health and General Practice, Norwegian University of Science and Technology, Trondheim, Norway; 5 Jena University Hospital, Friedrich-Schiller-University, Institute of General Practice and Family Medicine, Jena, Germany; 6 Institute of General Practice / Family Medicine, University Hospital of LMU Munich, München, Germany

**Keywords:** Patient activation, Empowerment, Self-management, International comparison

## Abstract

**Background:**

To allow better assessment of patients’ individual competencies for self-management, the Patient Activation Measure (PAM) has been developed in the USA. Because the American studies have shown the PAM to be a valuable tool, several European countries have translated the instrument into their native languages (Danish, Dutch, German, Norwegian). The aim was to compare the psychometric properties in studies from the different countries and establish whether the scores on the PAM vary between the studies.

**Methods:**

Data from the four separate studies were subjected to the same data cleaning procedures and statistical analyses. The psychometric properties of the instruments were established with measures of data quality and scale structure. The mean patient activation score and distribution across four predefined activation levels were described and the differences between the four studies were tested with ANOVA (unadjusted and adjusted) followed by a post-hoc Tukey HSD test and the Pearson chi-squared test respectively.

**Results:**

The total N of the four studies was 5184. The percentage of missing values was low in all datasets, confirming the good quality of the datasets. Factor analyses revealed moderate to strong factor loadings on the first factor in all datasets. Cronbach’s α was high for all version, ranging from .80 (German) to .88 (Dutch). Item-rest correlations varied between .32 and .66, indicating a moderate to strong correlation of the individual items to the sum scale.

Both the mean PAM score and the distribution across activation levels differed between the four datasets. After adjustment of the PAM score, patients in Norway in particular had a higher patient activation level.

**Conclusions:**

The European translations of PAM-13 (into Danish, Dutch, German and Norwegian) resulted in four instruments with good psychometric capabilities for measuring patient activation. The mean PAM score and the distribution across activation levels differed between the four datasets.

## Background

As people are generally living longer and medical treatment possibilities have improved, numbers of chronically ill patients are rising. In 2012, 29 % of men and 33 % of women in Europe reported having one or more chronic diseases or long-term health problems [1]. These percentages are higher for the elderly and for people with lower levels of education. The growing numbers of chronically ill people are placing a heavy burden on the healthcare system resources of western countries, both with respect to costs and manpower.

Patients with chronic illnesses have to cope with their disease and its consequences every day. This is also referred to as self-management. Lorig defines self-management as ‘learning and practicing skills necessary to carry on an active and emotionally satisfying life in the face of a chronic condition’ [2]. She discerns three different types of self-management tasks: medical management, role management, and emotional management [3]. Patients who are more active in self-management generally have better health outcomes and report a better health-related quality of life [4–6]. There are also studies that show an association between better self-management and lower healthcare costs [6, 7]. The importance of self-management is widely recognised, but nevertheless many barriers exist in the implementation of self-management behaviour. These barriers can be categorised as individually based (e.g. low skills, motivation and self-confidence, emotional distress), relationship-based (e.g. lack of social support) and environmentally based barriers (e.g. negative stimuli for healthy behaviour in society) [8].

To allow better assessment of patients’ individual competencies for self-management, Hibbard et al. developed and tested the Patient Activation Measure (PAM) in 2004 [9, 10]. The instrument was first developed in a longer version (22 questions) and slightly adapted later to a shorter version with 13 items. This version of the PAM is currently being used. Patient activation is defined as ‘an individual’s knowledge, skills, and confidence for managing their health and healthcare’ [10]. The PAM has been extensively validated and researched, although this was predominantly done in the USA. A recent report by Hibbard and Gilburt [11] provides an overview of the concept and measurement instrument, describes the positive relationship between patient activation and several health behaviours and outcomes, illustrates how the measurement instrument can be used and states the considerations as to why and how the PAM should be implemented.

The PAM scores have been used to divide people into one of four progressively higher activation levels, from passive and lacking knowledge and skills in dealing with health and healthcare in level 1 to active, generally well-informed and competent in level 4 (Table 6) [11, 12]. Several American studies have shown that care tailored to a patient’s activation level as measured with the PAM resulted in improved values on clinical indicators, better adherence to medication regimens and a reduction in hospitalisations and emergency department visits [13, 14]. Also, patient activation appeared to be modifiable and increases in activation have been found to be followed by improvement in self-management behaviour [14, 15].

Because the studies in the USA have shown the PAM to be a valuable tool, researchers in several European countries have translated the instrument into their native languages and validated it in a European setting [16–19]. In this article, data from different studies using the Danish, Dutch, German and Norwegian versions of the PAM are compared with each other and where possible with the original data from the USA.

The main aim of this study is to compare the psychometric properties of PAM in surveys from the different countries and compare the mean PAM score and the distribution between the four PAM levels between the studies.

## Methods

### The Patient Activation Measure

The 13-item version of the PAM was used in all four European studies [10]. The 13 items are: (1) When all is said and done, I am the person who is responsible for taking care of my health; (2) Taking an active role in my own health care is the most important thing that affects my health; (3) I am confident I can help prevent or reduce problems associated with my health; (4) I know what each of my prescribed medications do; (5) I am confident that I can tell whether I need to go to the doctor or whether I can take care of a health problem myself; (6) I am confident that I can tell a doctor concerns I have even when he or she does not ask; (7) I am confident that I can follow through on medical treatments I may need to do at home; (8) I understand my health problems and what causes them; (9) I know what treatments are available for my health problems; (10) I have been able to maintain (keep up with) lifestyle changes, like eating right or exercising; (11) I know how to prevent problems with my health; (12) I am confident I can figure out solutions when new problems arise with my health: (13) I am confident that I can maintain lifestyle changes, like eating right and exercising, even during times of stress. All items have five possible responses with scores ranging from 0 to 4; (1) disagree strongly, (2) disagree, (3) agree, (4) agree strongly or (0) not applicable. We calculated the mean score for the PAM items, leaving out items left blank or deemed not applicable by the respondents. Thus if the respondent had answered e.g. 11 items, the average of these was calculated. Participants who filled out fewer than seven questions were excluded, as were participants who answered all items with ‘disagree strongly’ or ‘agree strongly’ because these response sets were considered highly unlikely. The mean score was transformed into a standardised activation score ranging from 0 to 100 (the PAM score), based on a conversion table provided by the developers for data collected before 2014. The PAM score was then converted into one of the four levels of patient activation [12].

### Studies included

In the Danish study (*N* = 328), patients with different types of dysglycaemia were studied. In the Netherlands, the study included patients with a chronic illness who are taking part in an ongoing Dutch National Panel of People with Chronic Illness or Disability (NPCD) (*N* = 1829). In the study in Germany, adult patients from multiple primary care centres (in Germany, Austria and Switzerland) were included (*N* = 488). The Norwegian study included a sample of patients attending a range of different self-management courses provided by hospitals for persons with chronic conditions (*N* = 2539). The results from the USA were taken from the 13-item validation study [10].

The demographic characteristics of the four samples are shown in Table 1. The respondents of the German sample were relatively young (average age 54) and the Danish relatively old (average age 65). There were relatively more men in the Danish sample than the other groups and there were some differences in self-reported health status with the Norwegian and Dutch sample reporting worse self-reported health.

**Table 1:** Demographic characteristics

Variable	Danish	Dutch	German	Norwegian	USA
Mean age (SD)	62.2 (7.1)	58.9 (15.9)	54.4 (16.5)	58.3 (12.5)	Range 45 - 97
Age groups (N)	328	1829	488	2539	1469
Younger than 45	3 (1 %)	378 (21 %)	132 (27 %)	353 (14 %)	0 (0 %)
45–54 years	48 (15 %)	239 (13 %)	110 (23 %)	485 (19 %)	512 (38 %)
55–64 years	143 (44 %)	476 (26 %)	96 (20 %)	800 (32 %)	398 (28 %)
65–74 years	128 (39 %)	428 (23 %)	87 (18 %)	583 (23 %)	290 (20 %)
75 and older	6 (2 %)	308 (17 %)	63 (13 %)	318 (13 %)	188 (15 %)
Sex (N)	328	1829	487	2284	1469
Male	183 (56 %)	800 (44 %)	240 (49 %)	961 (42 %)	543 (37 %)
Female	145 (44 %)	1029 (56 %)	247 (51 %)	1323 (58 %)	926 (63 %)
Health status (N)	320	1823	480	2431	
Excellent	12 (4 %)	36 (2 %)	80 (17 %)	31 (1 %)	255 (18 %)
Very good	79 (25 %)	192 (11 %)	188 (39 %)	277 (11 %)	465 (31 %)
Good	181 (57 %)	1005 (55 %)	121 (25 %)	983 (40 %)	411 (28 %)
Fair	42 (13 %)	525 (29 %)	78 (16 %)	867 (36 %)	236 (16 %)
Poor	6 (2 %)	65 (4 %)	13 (3 %)	273 (11 %)	102 (7 %)

### Statistical analyses

Data from the four separate studies were combined and subjected to the same procedures of data cleaning and data management. The total N of the four studies was 5184. Given that the same data cleaning procedures were now used throughout, there are minor differences between the results presented in this article and in the previous Danish [16] and German [18] publications.

We then analysed the data from each country separately. The psychometric properties of the instruments were established with measures of data quality (percentage of missing data and of ‘non applicable’ answers) and scale structure (factor analyses, Cronbach’s α, item rest correlations). We performed a principal factor analysis (unrotated) for each dataset to see whether the PAM consists of one factor as in the original study. The cut-off point for the eigenvalue was ≥ 1. We then determined Cronbach’s alpha and the item rest correlations for the factors. We considered a scale as being sufficiently internally consistent if Cronbach’s alpha was ≥ 0.70. An item rest correlation of ≥ 0.50 was considered strong, *r* ≥ 0.30 moderate and *r* ≥ 0.10 weak.

The mean PAM score and distribution for each level were described for each country. With the Pearson chi-squared test, we determined whether the distribution for each level varied between the four datasets. We performed an ANOVA (unadjusted and adjusted for age, sex and self-reported health status) followed by a post-hoc Tukey HSD test to determine whether the mean PAM score differed. All analyses were performed using Stata 12.1.

## Results

### Psychometric properties

The mean scores along with the percentage of missing data and of ‘non applicable’ answers from the four studies on the 13 items are shown in Table 2. In the German study, the answer category ‘not applicable’ (NA) was not used. In general a very low percentage of missing values was reported, between 0.0 and 3.2. This confirms the good quality of these datasets.

**Table 2:** Scores on PAM items

PAM item no	Danish			Dutch			German			Norwegian		
	Mean (SD)	% MV	% NA	Mean (SD)	% MV	% NA	Mean (SD)	% MV	% NA	Mean (SD)	% MV	% NA
1	3.60 (0.53)	0.0	0.6	3.32 (0.71)	1.0	1.7	3.65 (0.57)	0.8	-	3.67 (0.57)	0.6	0.1
2	3.48 (0.57)	0.3	0.3	3.20 (0.69)	3.2	12.9	3.55 (0.59)	1.2	-	3.82 (0.47)	0.3	0.4
3	3.49 (0.59)	0.6	0.9	3.10 (0.73)	1.5	5.1	3.56 (0.59)	0.2	-	3.24 (0.70)	1.3	0.5
4	3.14 (0.66)	3.1	17.4	3.20 (0.66)	0.4	5.1	3.74 (0.52)	0.6	-	3.24 (0.78)	1.1	10.2
5	2.99 (0.72)	0.6	8.5	3.24 (0.60)	0.5	0.7	3.39 (0.67)	0.6	-	3.20 (0.72)	0.8	1.3
6	3.26 (0.66)	0.3	4.6	3.29 (0.61)	0.9	0.9	3.64 (0.60)	0.2	-	3.50 (0.67)	0.5	0.4
7	3.37 (0.55)	0.3	2.7	3.30 (0.57)	1.6	17.6	3.01 (0.91)	1.8	-	3.58 (0.65)	1.0	5.5
8	3.28 (0.59)	0.0	7.6	3.23 (0.67)	0.9	2.7	3.08 (0.78)	0.6	-	3.15 (0.80)	0.4	0.8
9	3.02 (0.67)	0.3	17.1	3.10 (0.67)	1.4	4.3	2.90 (0.85)	1.4	-	2.81 (0.84)	0.7	1.1
10	3.00 (0.68)	0.6	5.5	3.03 (0.66)	1.8	12.9	3.15 (0.70)	1.0	-	3.26 (0.69)	1.1	3.4
11	3.08 (0.57)	0.6	8.8	2.95 (0.70)	1.0	6.8	3.22 (0.66)	1.0	-	2.99 (0.82)	1.1	0.8
12	2.94 (0.60)	0.3	16.5	2.62 (0.75)	1.2	8.0	2.97 (0.83)	0.6	-	2.82 (0.75)	3.0	1.7
13	2.83 (0.74)	0.0	11.9	2.84 (0.70)	1.0	7.4	2.90 (0.80)	0.4	-	3.08 (0.73)	2.7	1.3

%MV = percentage of missing values

%NA = percentage not applicable; category not used in German studies

Explorative factor analysis led to the identification of one factor with an eigenvalue of ≥ 1 in the Dutch, German and Norwegian versions of the questionnaire (as in the original American one). In the Danish data, two factors with eigenvalues of ≥ 1 were identified. Items 1, 2 and 3 of the PAM-13 had a higher loading on factor 2 than factor 1. However, these items also had a sufficient factor loading (≥0.40) on factor 1, as did the remaining items. Therefore, as in the other studies, we determined the internal consistency of the first factor consisting of all 13 items in the Danish study.

Cronbach’s α for all four versions of the PAM was similar and high, varying from .80 (German) to .88 (Dutch) (Table 3). This confirms the good internal consistency of the instruments. Item-rest correlations vary between .32 and .66 in all versions of the PAM, indicating a moderate to strong correlation of the individual items to the sum scale.

**Table 3:** Factor analyses and reliability

Test	Danish		Dutch	German	Norwegian
	Factor 1	Factor 2^a^	Factor 1	Factor 1	Factor 1
Percentage of explained variance	69.5 %	24.4 %	85.4 %	90.8 %	96.7 %
Factor loadings	0.41–0.65	|0.01|–|0.63|	0.47–0.72	0.32–0.63	0.44–0.64
Cronbach’s alpha	0.86		0.88	0.80	0.84
Item-rest correlations	0.44–0.60		0.46–0.66	0.32–0.57	0.37–0.60

^a^The first three items had a higher loading for factor 2 than factor 1, but the loading for factor 1 was still >0.40. We therefore only performed the reliability analyses for factor 1 containing all 13 items

### Patient activation scores

The mean scores of the items in all versions of the PAM varied between 2.62 and 3.82 (Danish 2.83–3.60, Dutch 2.62–3.32, German 2.90–3.74, Norwegian 2.81–3.82, Table 2). We looked at the distribution of patients across PAM levels. This distribution differed significantly between the four datasets (*p* < 0.001). The percentage of patients in the lowest two (i.e. least activated) levels of the PAM (see Table 6) was especially high in the Netherlands (37 %). In the other countries, 18 % (German-speaking group) to 22 % (Danish-speaking group) of the patients belonged to these two levels (Table 4).

**Table 4:** Mean patient activation score and distribution per level

PAM score and activation level	Danish (*n* = 328)	Dutch (*n* = 1829)	German (*n* = 488)	Norwegian (*n* = 2539)	USA
Mean PAM score	64.1	61.2	67.2	66.3	61.9
Activation level					
Level 1	29 (9 %)	313 (17 %)	45 (9 %)	283 (11 %)	
Level 2	41 (13 %)	357 (20 %)	44 (9 %)	235 (9 %)	
Level 3	125 (38 %)	588 (32 %)	127 (26 %)	640 (25 %)	
Level 4	133 (41 %)	571 (31 %)	272 (56 %)	1381 (54 %)	

We have presented the comparison between the mean PAM scores of the four versions of the PAM in Table 5, both unadjusted and adjusted for age, sex and self-reported health.

**Table 5:** Comparison of mean activation score (standard error in parentheses) between countries with and without taking account of age, sex and self-reported health status

PAM score	Danish	Dutch	German	Norwegian	F
PAM unadjusted	64.1 (0.80)	61.2 (0.34)	67.2 (0.66)	66.3 (0.29)	49.66*
PAM adjusted	62.1 (0.79)	61.4 (0.33)	63.7 (0.68)	67.4 (0.30)	63.42*

*p < 0.001

While the unadjusted mean PAM score in the Netherlands is similar to the one in the USA, the PAM scores in the other countries were higher. Furthermore the PAM scores between the different studies differed significantly (*p* < 0.05) except between the German and the Norwegian data (*p* = 0.627).

When adjusted for age, sex and self-reported global health, all PAM scores differ significantly from each other, except between the Danish and the Dutch data (*p* = 0.828) and the Danish and the German data (*p* = 0.441). This means that the Norwegian patients had a higher activation level than all other groups and that the German-speaking patients had a higher activation level than the Dutch patients.

## Discussion

The results of this study confirmed the results of the earlier published studies [16–19] that the translations of the PAM-13 (into Danish, Dutch, German and Norwegian) resulted in four instruments with good psychometric capabilities for measuring patient activation. The psychometric properties of the PAM were similar across the different studies.

The unadjusted mean scores on the PAM did differ between the four studies. On average, the Dutch patients had the lowest mean PAM score at 61.2, while the German-speaking patients had the highest mean PAM score at 67.2. Danish and Norwegian patients were positioned in the middle. The differences between the mean scores on the four questionnaires can partly be explained by the variation in the samples and recruitment procedures. While Denmark, the Netherlands and Norway administered their questionnaires to older, chronically ill patients, the German-speaking respondents were younger (50 % < 55 years) and recruited at primary care centres. After correcting for age, sex and self-reported health, the mean scores still differed between the four versions of the PAM. But now, the Norwegian patients had a higher PAM score than the other three groups.

The distribution across the four PAM levels was somewhat different, with the percentage of patients in the lowest two levels of the PAM (see Table 6) being especially high in the Netherlands (37 %). It might be that the distribution is different due to the fact that the countries included here have different cultures and healthcare systems. Using focus groups, Hibbard et al. established that people in the four patient activation levels differed in terms of self-management behaviour [9]. On the basis of their research, the developers of the PAM established the cut-off points of the four groups [12]. When translating and validating the PAM in another language and country, it is also sensible to examine whether the four levels are also associated with different behaviours or that the cut-off points should be placed elsewhere. This might have important implications when the PAM score is used to tailor healthcare to a patient’s activation level or to improve someone’s activation level.

**Table 6:** The four levels of patient activation

Level 1	Individuals tend to be passive and feel overwhelmed by managing their own health.
	They may not understand their role in the care process.
Level 2	Individuals may lack the knowledge and confidence to manage their health.
Level 3	Individuals appear to be taking action but may still lack the confidence and skill to support their behaviours.
Level 4	Individuals have adopted many of the behaviours needed to support their health but may not be able to maintain them in the face of life stressors

The fact that a lower (poorer) activation score was found in the Netherlands is striking, given that the Netherlands scored best in a European comparative study on health literacy in eight countries (HLS-EU), with a percentage of 28.7 % people with limited health literacy, whereas this same percentage was 46.3 in Germany and 56.4 in Austria [20]. Denmark and Norway were not included in that study. Taken together, it seems that the people in the Netherlands perform better than the populations in these German-speaking countries with respect to accessing, understanding, appraising and applying health-related information (the HLS-EU definition of health literacy) but that they are more likely to perceive themselves as lacking the psychosocial skills such as motivation and self-confidence (central parts of the PAM score), which are equally important for self-management, if not indeed more important. Earlier studies already demonstrated that the overlap between health literacy (when defined in a functional way) and patient activation is limited [21–23]. However, with a broader conceptualisation of health literacy that includes psychosocial and contextual variables, as is being done in the Health Literacy Questionnaire (HLQ) [24], the overlap will inevitably increase.

The main limitation of this study is that it compares different studies with different inclusion criteria, making the samples different with respect to e.g. age and health status. This may have led to differences between the reported scores. However, even after adjustment for these variables, the majority of the variation between the scores remained.

Another limitation is the fact that the answer categories were different for the dataset of the German version (leaving out the ‘not applicable’ option). This might have led to somewhat higher mean scores on the German version of the PAM, thus exaggerating the differences.

The strength of this study is that we used the same methods for data cleaning and data management and statistical analyses for the data from these four European studies. We were therefore able to assess and test similarities and differences more accurately between the psychometric aspects of the instruments and between the scores.

In this study, we looked at psychometric properties such as data quality and scale structure. These are methods used in the classical test theory to get insights into the validity and reliability of an instrument. An interesting next step would be to use Item Response Theory (IRT) and Differential Item Function (DIF) analyses to assess whether different items present as more or less “difficult” to different people of different countries.

## Conclusions

The European translations of the PAM (into Danish, Dutch, German and Norwegian) resulted in four instruments with good psychometric capabilities for measuring patient activation. The mean PAM scores and the distribution across activation levels differed between the four datasets.

## Abbreviations

DIF: Differential Item Function
HLQ: Health Literacy Questionnaire
HLS-EU: Health Literacy Survey – Europe
HSD: Honest Significant Difference
IRT: Item Response Theory
PAM: Patient Activation Measure
USA: United States of America
